# Detection of Mycolactone A/B in *Mycobacterium ulcerans*–Infected Human Tissue

**DOI:** 10.1371/journal.pntd.0000577

**Published:** 2010-01-05

**Authors:** Fred Stephen Sarfo, Richard O. Phillips, Brian Rangers, Engy A. Mahrous, Richard E. Lee, Edward Tarelli, Kingsley B. Asiedu, Pamela L. Small, Mark H. Wansbrough-Jones

**Affiliations:** 1 Komfo Anokye Teaching Hospital, Kumasi, Ghana; 2 School of Medical Sciences, Kwame Nkrumah University of Science and Technology, Kumasi, Ghana; 3 University of Tennessee Health Science Center, Memphis, Tennessee, United States of America; 4 St. George's, University of London, London, United Kingdom; 5 World Health Organization, Geneva, Switzerland; London School of Hygiene and Tropical Medicine, United Kingdom

## Abstract

**Background:**

*Mycobacterium ulcerans* disease (Buruli ulcer) is a neglected tropical disease common amongst children in rural West Africa. Animal experiments have shown that tissue destruction is caused by a toxin called mycolactone.

**Methodology/Principal Findings:**

A molecule was identified among acetone-soluble lipid extracts from *M. ulcerans* (Mu)-infected human lesions with chemical and biological properties of mycolactone A/B. On thin layer chromatography this molecule had a retention factor value of 0.23, MS analyses showed it had an m/z of 765.6 [M+Na^+^] and on MS:MS fragmented to produce the core lactone ring with m/z of 429.4 and the polyketide side chain of mycolactone A/B with m/z of 359.2. Acetone-soluble lipids from lesions demonstrated significant cytotoxic, pro-apoptotic and anti-inflammatory activities on cultured fibroblast and macrophage cell lines. Mycolactone A/B was detected in all of 10 tissue samples from patients with ulcerative and pre-ulcerative Mu disease.

**Conclusions/Significance:**

Mycolactone can be detected in human tissue infected with Mu. This could have important implications for successful management of Mu infection by antibiotic treatment but further studies are needed to measure its concentration.

## Author Summary

Skin infection with bacteria called *Mycobacterium ulcerans* causes Buruli ulcer, a disease common in West Africa and mainly affecting children. *M. ulcerans* is the only mycobacterium to cause disease by production of a toxin. This lipid molecule called mycolactone diffuses from the site of infection, killing surrounding cells and, at low concentration, suppressing the immune response. The aim of this study was to show that mycolactone can be detected among lipids extracted from human *M. ulcerans* lesions in order to study its role in the pathogenesis of *M. ulcerans* disease. Lipids were extracted from skin biopsies and tested for the presence of mycolactone using thin layer chromatography and mass spectrometry. The extracts were shown to kill cultured cells in a cytotoxicity assay. Mycolactone was detected in both pre-ulcerative and ulcerative forms of the disease and also in lesions during antibiotic treatment but with reduced bioactivity, suggesting a lower concentration compared to untreated lesions. These findings indicate that there is mycolactone in affected skin at all stages of *M. ulcerans* disease and it could be used as a biomarker for monitoring the clinical response to antibiotic treatment.

## Introduction

*Mycobacterium ulcerans* (Mu) disease (Buruli ulcer) is common in humid rural tropical areas mainly in West Africa and predominantly affects children between 5 and 15 years of age [1]. The classic lesion is a painless nodule which breaks down centrally to form an ulcer with undermined edges. Histology shows clumps of acid fast bacilli in areas of subcutaneous fatty necrosis with acute and chronic inflammation remote from the necrotic areas. Granulomas are found in later lesions [2]. This histopathology led to the suggestion that Mu causes disease by secretion of a toxin which can destroy human tissue and inhibit the development of local inflammation [3]. Subsequently it was found that *M. ulcerans* culture filtrate could produce similar lesions after injection into guinea pig skin [4].

**Figure 1:**
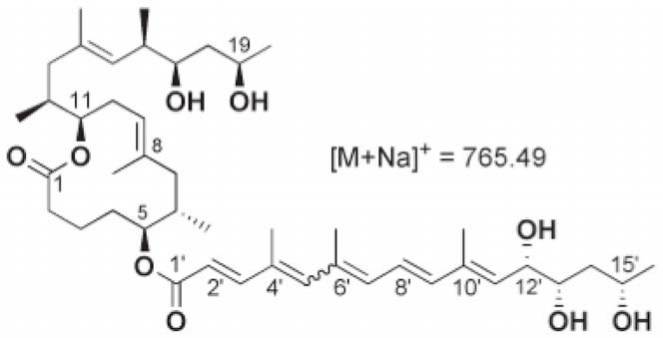
Chemical structure of mycolactone A/B showing the lactone core ring and polyketide side chains.

Initial attempts to isolate this substance were frustrated by low yields from cultures until the late 1990s when a toxin called mycolactone was partly purified and its chemical structure defined [5]. Subsequently mycolactone has been characterised as a 743 Da molecule consisting of a 12-membered ring macrolide with two polyketide derived side chains (figure 1) synthesised by giant polyketide synthases and polyketide modifying enzymes whose genes are carried on two identical copies of a 174 kb plasmid known as pMUM001 [6]. Mycolactone causes a cytopathic effect on mouse fibroblast L929 cells characterised by cytoskeletal rearrangement with rounding up and subsequent detachment from tissue culture plates within 48 hours. The toxin causes cell cycle arrest in the G0/G1 phase within 48 hours, proceeding to cell death by apoptosis after 72 hours [7].

Various elegant *in-vitro* and *in-vivo* studies in mice and guinea pigs have demonstrated that this polyketide toxin is central to the pathogenesis of *M. ulcerans* disease. George et al demonstrated that injection of 100 µg of mycolactone was sufficient to cause characteristic ulcers in guinea pig skin [7] and that histopathological changes could be detected with 10 µg. Although direct inoculation of mycolactone intradermally into guinea pigs caused necrotic lesions similar to those produced by the injection of live organisms, an isogenic toxin-negative mutant *M. ulcerans* was phagocytosed by macrophages and stimulated a typical mycobacterial inflammatory response, including granuloma formation. Chemical complementation of this mutant with mycolactone restored virulence [8]. Histologically these lesions showed significant apoptotic cell death [9], a feature which has been observed in human lesions [10]. Mycolactone has also been associated with vacuolar nerve tissue damage in mice and this observation may account for the painlessness of Buruli ulcer lesions [11].

The classic histological feature of human Buruli ulcer lesions is subcutaneous fatty necrosis with clumps of AFB in the absence of inflammatory cells. The necrosis is explained by cytotoxic properties of mycolactone but the paucity of inflammatory cells despite extensive skin damage may be due to its immunosuppressive properties. Mycolactone has been shown to inhibit the responses of macrophages and activated T-cells *in-vitro* [12], to inhibit phagocytosis by murine macrophages [13], to induce lysis of cultured macrophages after a transient intracellular growth [14] as well as to impair the production of TNF-alpha by these cells [15] and to inhibit induction of chemokine secretion by dendritic cells [16]. Therefore high concentrations of mycolactone in necrotic foci may kill cells by apoptosis or necrosis but its diffusion away from the centre of lesions where infiltrates of neutrophils and macrophages are found may serve to modulate the release of chemokines and cytokines by these inflammatory cells without killing them.

Despite the obvious role of mycolactone in virulence, the fact that the molecule is a lipid rather than a protein has made it difficult to study *in vivo*. The molecule has not been detected *in vivo* in human Buruli ulcer lesions making it impossible to answer questions regarding its distribution and stability. Although it has been assumed that mycolactone is present in all stages of infection, there has been no evidence to support this speculation. The aim of the present studies was therefore to identify mycolactone among lipids extracted from human skin infected with Mu.

## Materials and Methods

Ten patients with a clinical diagnosis of *M. ulcerans* disease were recruited at Tepa Government Hospital in the Ahafo Ano north district in the Ashanti Region of Ghana. After the patients had given informed consent, two 4 mm punch biopsies were obtained from the centre of non-ulcerated lesions or from the edge of the viable skin around ulcers. One biopsy was used to establish the diagnosis of Mu disease using microscopy, culture and PCR for the repeat sequence IS2404 of Mu as described elsewhere [17]. To detect Mu, homogenised tissue was stained by the Ziehl-Neelsen technique and 1 ml was decontaminated by the modified Petroff method for 10 minutes and inoculated onto Löwenstein-Jensen slopes. Cultures were incubated at 31°C and examined weekly for 6 months before they were discarded. DNA extraction was performed by the guanidinium thiocyanate diatoms technique [17] and PCR was performed targeting the 1S2404 insertion sequence as described elsewhere [17]. The other biopsy specimen was snap frozen in liquid nitrogen and stored at −70°C for lipid analysis.

### Extraction of lipids from human skin

Lipids were extracted from Buruli ulcer lesions using chloroform:methanol 2:1 (vol/vol) followed by a Folch extraction with 0.2 volumes water. Briefly, the punch biopsy was weighed and placed in a 1.5 ml green top matrix tube containing 500 µl of diatoms (Q-bio) and homogenised in extractant solution for 45 seconds at a power of 6.5 in a Fast Prep Ribolyser.The homogenate was transferred into a microfuge tube and allowed to stand for 30 minutes. After centrifugation at 10,000 g for 2 minutesthe organic phase was harvested. The organic phase was dried in a roto-evaporator and re-suspended in ice-cold acetone for 1 hour to precipitate phospholipids. Acetone soluble lipids (ASL) were harvested for mycolactone detection by thin layer chromatography, mass spectrometry and cytopathicity assays. For cytopathicity assays, half of the total volume of lipid extracts were dried under nitrogen gas and shipped to the University of Tennessee, U.S. where analyses were performed. The other half of tissue lipid extracts were analysed for cytotoxicity using an MTT assay, fluorescent microscopy for characterisation of cell death and TNF-alpha induction assays at St. George's, University of London, U.K.

To optimise the extraction technique and determine the limit of detection, 100 µg, 10 µg, 1 µg and 0.1 µg quantities of mycolactones were used to spike approximately 100 mg of healthy skin tissue. Healthy human skin was obtained during excision of a lipoma with patient permission. Mycolactone was extracted from spiked skin samples as described above.

### Preparation of mycolactone A/B from bacterial extracts

Mycolactone was prepared from *M. ulcerans* extracts as previously described [5]. Briefly, *M. ulcerans* cultures were grown in Middlebrook M7H9 broth with OADC supplement until early stationary phase. Bacteria were harvested by centrifugation and mycolactone was extracted from dried bacterial pellets using chloroform:methanol 2:1 followed by Folch extraction with 0.2 volumes water. Acetone-soluble lipids (ASL) highly enriched for mycolactone were obtained by drying lipids under nitrogen and enriched for mycolactone by precipitating phospholipids with ice cold acetone. Purified mycolactone was obtained from ASL by centripetal chromatography using a Harrison chromototron (Palo Alto, CA). Samples were assessed by mass spectroscopic analysis to validate purity.

### Thin layer chromatography

Purified mycolactone A/B was applied to a silica thin layer chromatography (TLC) plate and analysed using chloroform-methanol-water (90:10:1 vol/vol/vol) as a solvent system. Lipid bands were visualised under UV light and by oxidative charring with ceric sulphate-ammonium molybdate in 2M sulphuric acid. Serial dilutions of purified mycolactone from 125 µg/ml to 1.4 µg/ml were spotted onto TLC plates to determine the detection limit for the system.

20 µl of acetone soluble lipids from spiked skin samples, negative controls and infected human tissues were analysed by TLC to detect the presence of mycolactone bands.

### Electron spray ionisation mass spectrometry (MS)

Acetone soluble lipids were dissolved in ethanol and directly perfused into an electrospray ionization source on a Bruker Esquire 2000 mass spectrometer using a Cole Palmer 74900 series syringe pump. The electrospray MS conditions were initially optimized to a mycolactone standard before applying them to the lipid extracts. The electrospray MS conditions were: infusion rate 1,000 µl/h; nebulizer pressure 30 lb/in^2^; dry gas flow 10 l/min; dry temperature 320°C; capillary voltage −4,000 V; end plate offset −500 V. Detection of mycolactone was determined by the presence or absence of ions characteristic of mycolactone: the more abundant sodium adduct [M+Na]^+^ (m/z 765.5); the protonated molecular ion [M+H]^+^ (m/z 743.5), and the dehydrated protonated molecular ion [M+H - H_2_O]^+^ (m/z 725.5). ESI-MS/MS analyses were performed on the m/z 765.5 component for the characteristic fragmentation pattern comprising ions corresponding to the core lactone and the polyketide side chain respectively.

### Cytopathic effect assays

L929 murine fibroblasts were maintained in Dulbecco's modified Eagle's medium with 5% foetal calf serum in tissue culture flasks and incubated in 5% carbon dioxide at 37°C. ASL samples which had been dried under nitrogen were dissolved in 50 µl absolute ethanol. 2.5 µl (5%) or 5 µl (10%) of the resultant solution was added to cells in a 96-well tissue culture plate to determine cytopathic effect (CPE). CPE was defined as the minimal concentration of ASL per millilitre necessary to produce 90% cell rounding in 24 h and loss of the monolayer by 48 h.

Cytotoxicity of ASL from infected human tissues was further assessed by the ability of mycolactones to inhibit mitochondrial succinate dehydrogenases of human embryonic lung fibroblasts (HELF) from reducing dimethylthiazolyl diphenyl tetrazolium bromide (MTT) dye to form purple formazan crystals. Induction of cell death was assessed by staining these cells with a dye mix of ethidium bromide and acridine orange after treatment of HELF cells with mycolactones positive controls, acetone soluble lipids from infected patient samples and negative controls comprising ASL a lipoma lesion, ethanol and untreated wells.

Briefly HELF cells (kindly donated by Dr. Kay Capaldi) were maintained in Dulbecco's modified Eagle's medium supplemented with 10% foetal calf serum and 2 mM L-glutamine in the presence of penicillin and streptomycin 100 mU/ml and 100 mg/ml respectively and incubated in 5% carbon dioxide at 37°C. For cytotoxicity assays proliferating HELF cells were seeded at a density of 10^5^/well in microtitration plates overnight. ASL from lesions, positive controls and negative controls were dissolved in 100 µl of absolute ethanol of which 5 µl was used to treat HELF cells in quadruplicates. After 48 h incubation, 20 µl of 5 mg/ml of MTT (Sigma) was added to each well and incubated for a further 4 h for purple coloured formazan crystals to develop following which 100 µl of detergent solution of isopropanol: HCl (2N) in a ratio of 49∶1 was used to dissolve formazan crystals for spectrophotometric quantification in multiplate well reader at 570–690 nm.

To characterise the pattern of cell death caused by mycolactones in patient samples HELF cells were stained with 8 µl of a combination of 100 µg/ml of ethidium bromide (Sigma) and 100 µg/ml of acridine orange (Sigma) (EB/AO) dye mix in microtitration plates as previously described [18]. Cells were observed with a DM1 6000B Leica fluorescent microscope and images taken at x10 magnification.

To confirm the immunosuppressive properties of mycolactones extracted from human lesion we measured release of TNF-α by J774 macrophages pre-treated with ASL from patient lesions after stimulation with lipopolysaccharide (LPS). Briefly, J774 macrophages (kindly donated by Dr. Rajko Reljic) were maintained in Dulbecco's modified Eagle's medium supplemented with 10% foetal calf serum and 2 mM L-glutamine in tissue flasks and incubated in 5% carbon dioxide at 37°C. Proliferating macrophages were seeded at a density of 10^5^ in 96-well microtitration plates and allowed to adhere to culture plates over 4 h. Cells were exposed to 5 µl of ASL from lesions or to purified mycolactones as a positive control for 6 h and stimulated with 0.5 µg/ml of LPS (Sigma) with a 16 h incubation in 5% CO_2_ at 37°C. Samples were analysed in quadruplicates and experiments were repeated twice. Supernates were harvested and TNF-α quantities assayed in duplicate with a Quantikine ELISA kit (R & D Systems) according to the manufacturer's protocol. The limit of detection of TNF-α was 23.4 pg/ml. Viability of macrophages after cytokine induction assays was assessed with the MTT assay previously described.

### Statistical analysis

Student's t-test was used to test for significance by comparing cytotoxicity and TNF-alpha release by cultured cell lines treated with lipid extracts from patient samples and negative controls with a p<0.01 considered significant. The Mann-Whitney U test was used to compare TNF-alpha release by macrophages treated with lipid extracts from untreated and antibiotic treated lesions with p<0.05 considered statistically significant.

### Ethical review

The study protocol was approved by the ethics review committees at the School of Medical Sciences, Kwame Nkrumah University of Science and Technology, Kumasi, Ghana, and St. George's Hospital in London, United Kingdom. Informed consent was obtained orally since the patients were illiterate and consent was given by thumb print as approved by the ethics committees.

## Results

### Patient characteristics

Table 1 shows the characteristics of the 10 patients with PCR-confirmed *M. ulcerans* infection whose mean age was 17 years. There were 5 nodules, 4 oedematous lesions, 3 of which had ulcerated, and 1 ulcer without oedema. Five patients had received antibiotic therapy consisting of intramuscular streptomycin 15 mg/kg daily and oral rifampicin 10 mg/kg daily, 4 for 4 weeks and one for 6 weeks. Four out of 5 untreated lesions were culture positive and 3 out of 5 lesions were also culture positive after 4 or 6 weeks of treatment as shown in Table 1.

**Table 1:** Patient characteristics, diagnostic data and treatment status.

Code	Age(years)	Sex	Lesion Type	A.F.B.	Culture on L.J.	P.C.R.	Treatment Status*
7	10	F	oedema with ulcer	negative	positive	positive	untreated
8	18	F	nodule	negative	positive	positive	4 weeks antibiotics
9	16	M	oedema with ulcer	negative	positive	positive	untreated
10	15	M	oedema with ulcer	+++	negative	positive	untreated
11	9	F	nodule	negative	positive	positive	untreated
12	32	M	oedema	negative	negative	positive	6 weeks antibiotics
13	16	F	nodule	negative	negative	positive	4 weeks antibiotics
14	11	M	nodule	negative	positive	positive	untreated
15	25	F	nodule	++	positive	positive	4 weeks antibiotics
16	18	M	ulcer	+++	positive	positive	4 weeks antibiotics

*: Antibiotic treatment was with streptomycin injection15 mg/kg and oral rifampicin 10 mg/kg daily.

### Detection of mycolactone A/B in human skin lesions

**Figure 2:**
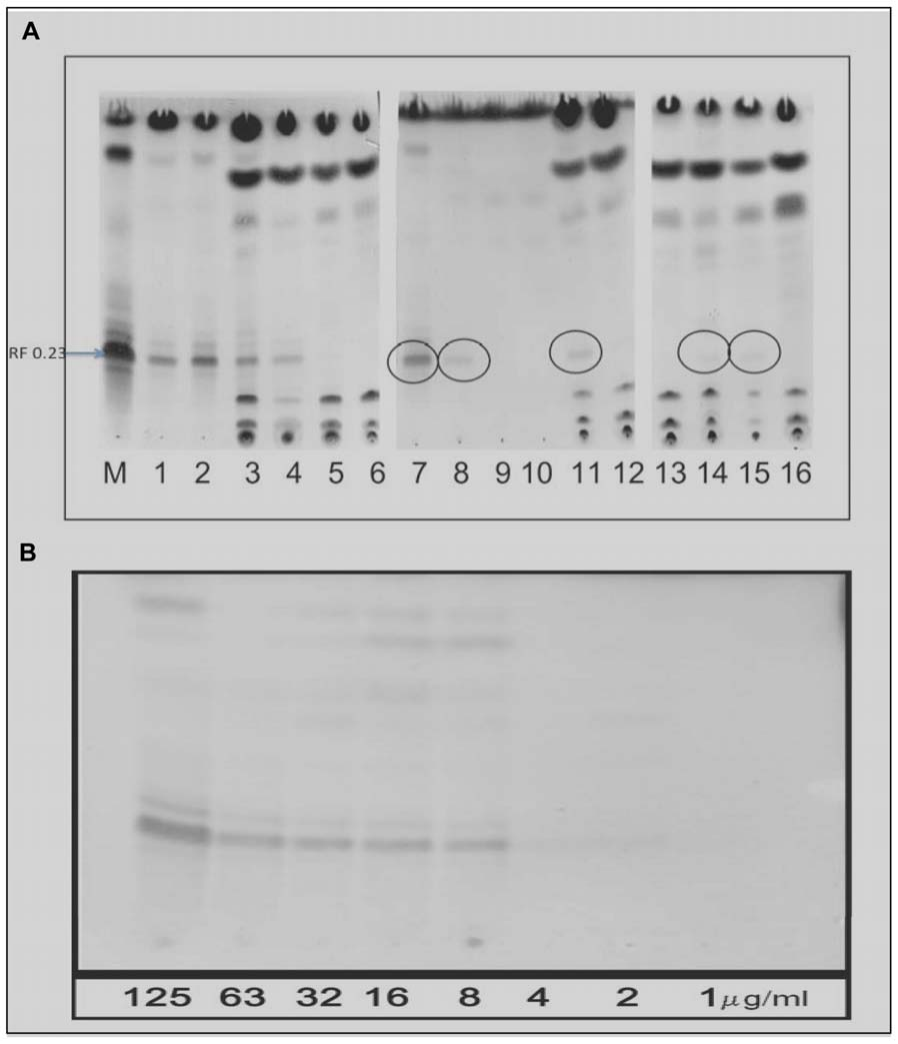
Detection of mycolactone A/B by thin layer chromatography. A. 20 µl of two-fold serial dilutions of mycolactone A/B at concentrations from 125 to 1 µg/ml were spotted and examined under UV-light and by oxidative charring. The detection limit on TLC was at a concentration of 2–8 µg/ml (8 µg corresponding to 160 ng of mycolactone A/B). B. Each track represents one sample. M is purified mycolactone A/B; tracks 1 and 2 are positive controls with100 µg of purified mycolactone; tracks 3 and 4 are samples extracted from human skin spiked with 100 µg of purified mycolactone; tracks 5 and 6 are negative controls from healthy human skin; tracks 7 to 16 are extracts from infected human skin samples. Mycolactone A/B was the predominant UV-active band with an Rf of 0.23 in positive controls and in ASLs from infected human skin. There were perceptible signals from patients 7, 8, 11, 14 and 15 whereas samples from 9, 10, 12, 13 and 16 were below the detection limit.

Using TLC, purified mycolactone A/B yielded a major UV active band at a retention factor (Rf) value of 0.23 and two minor bands. The detection limit of mycolactone on TLC was 2–8 µg/ml as shown in figure 2A. Figure 2B shows that acetone soluble lipids from 5 lesions had detectable bands at Rf 0.23 which were UV active and corresponded to the purified mycolactone A/B ladder M. These bands were from 1 untreated oedematous BU lesion with ulceration, 2 untreated nodules and 2 antibiotic treated nodules. All those with detectable mycolactone signals on TLC were also culture positive. However 5 other lesions did not show perceptible bands on TLC plates. Three of these patients were on antibiotics while the other 2 patients had untreated oedematous lesions.

**Figure 3:**
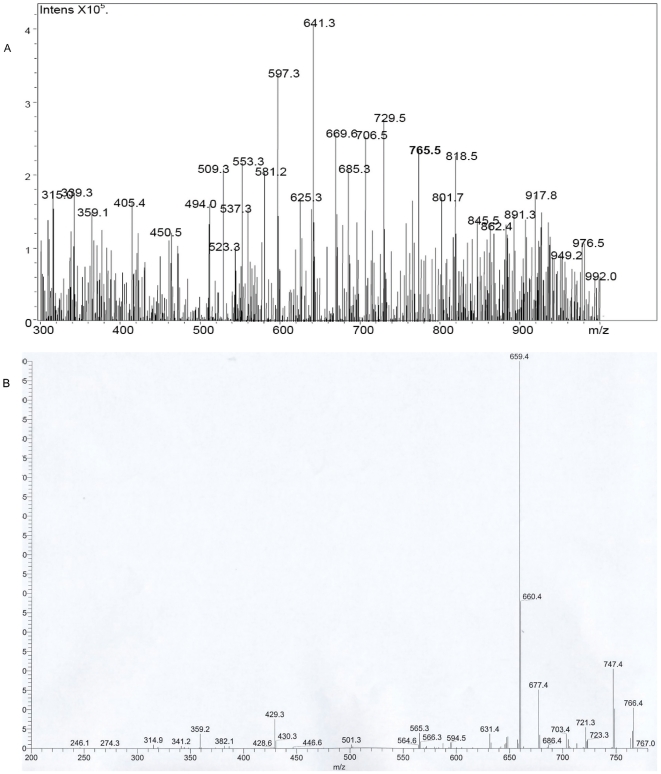
Detection of mycolactone by mass spectrometry. A. MS analysis of ASL from Mu infected human skin showing a molecule with m/z 765.5 which represents the sodium adduct of mycolactone A/B [M+Na^+^]. B. MS-MS analysis of this ion produced the core lactone ring of mycolactone with m/z 429.4 and the polyketide side chain of mycolactone A/B with m/z 359.2.

Mass spectral analysis of acetone soluble lipids from infected human lesions under microspray conditions revealed the presence of an ion with mass to charge ratio (m/z) of 765.5 [M+Na^+^] which corresponded to that of mycolactone A/B (Figure 3A). Fragmentation of this ion yielded ions with m/z 429.3 corresponding to the conserved core lactone ring present in all mycolactone congeners and with m/z of 359.2 corresponding to the polyketide side chain of mycolactone A/B. In addition ions were also observed at m/z 341.2, 565.3, 579.4, 659.4, 677.4, 707.4, 721.3, 747.4 and 766.4 (Figure 3B).

Mycolactone A/B was detected in acetone soluble lipids from all 10 patients by mass spectrometry. Using mycolactone 100 µg, 10 µg, 1 µg and 0.1 µg to spike approximately 100 mg of healthy skin tissue, mycolactone signals could be detected on MS from those spiked with 100 µg and 10 µg but not at lower concentrations.

### Cytopathicity assays of human acetone soluble lipids

Mycolactone exhibits a characteristic cytotoxic phenotype which includes cell rounding at 10 hours, cell cycle arrest at 36 hours and apoptotic cell death at 72 hours. We studied the effects of ASL on L929 murine fibroblasts without purifying mycolactone. This was justified since previous work has shown that the cytotoxicity of lipids obtained by chloroform∶methanol 2∶1 extraction is entirely due to the presence of mycolactone [7]. The classic cytotoxic phenotype of mycolactones was observed within 48 h upon treatment of murine fibroblasts with 5% ASL from infected Mu lesions. However, using 10% ASL, a high mycolactone concentration phenotype characterised by osmotic swelling of cells with eccentric nuclei was observed at 24 hours as previously described by Adusumilli et al [8] in all 10 patient samples analysed (data not shown).

**Figure 4:**
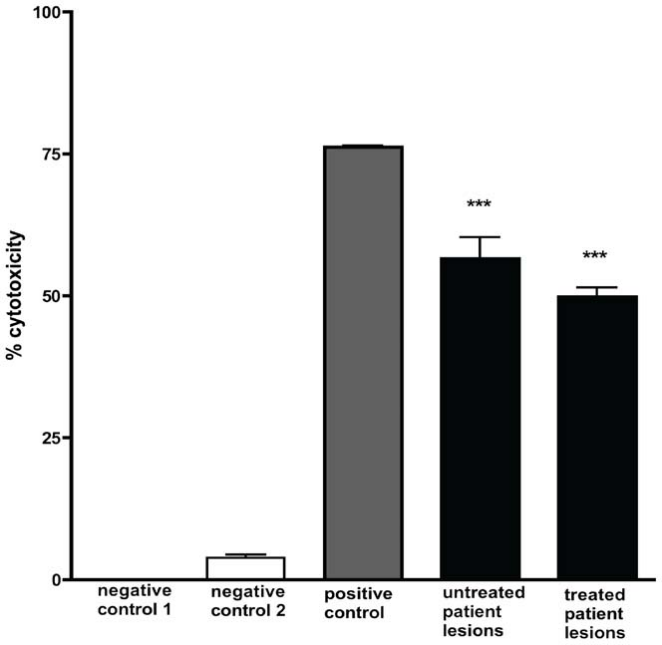
Cytotoxicity of ASL from human Mu lesions on human embryonic lung fibroblasts. Cytotoxicity after 48 h culture was assessed using an MTT assay. Negative control 1 is untreated cells, negative control 2 is ASL from uninfected skin. Positive control was purified mycolactone at a concentration of 5 µg/ml. Significant cytotoxicity was observed with all patient samples with ***p<0.001 compared to negative control 1. The apparent difference in percentage cytotoxicity between 5 untreated and 5 antibiotic treated lesions was not statistically significant. HELF cells were treated in quadruplicates and cytotoxicity determined in at least 2 independent experiments. Data are shown as a percentage of untreated cells (negative control 1). Error bars are ±SEM of duplicate assays.

**Figure 5:**
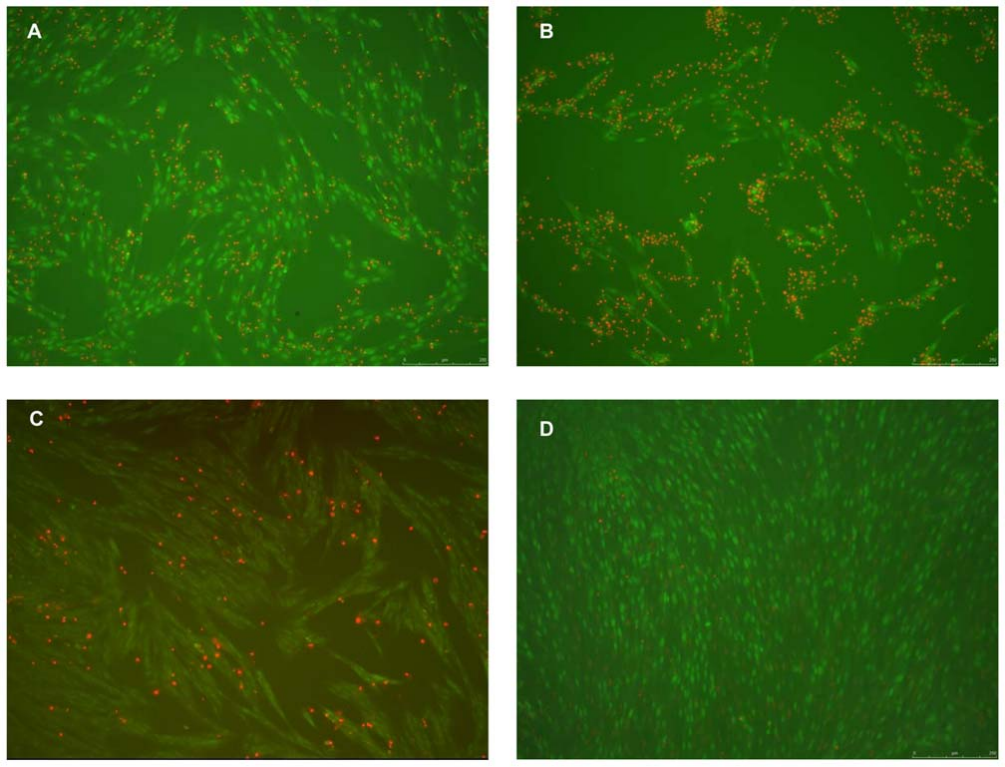
Cytotoxicity of mycolactone on HELF cells. A and B show the effect of ASL from Mu infected skin on HELF cells after 48 h with detachment of cells from culture plate, numerous apoptotic cells (orange nuclei stained with acridine orange) and few live spindle shaped cells (green nuclei with ethidium bromide). A and B represent ASL from a nodular lesion and an ulcerative lesion respectively. C shows the effect of purified mycolactone at a concentration of 5 µg/ml and D is a negative control demonstrating the effect of ASL from uninfected human skin. Pictures taken at x10 with a DM1 6000B Leica fluorescent microscope.

In MTT based cytotoxicity assays, ASL from Mu infected lesions significantly inhibited mitochondrial succinate dehydrogenases of human embryonic lung fibroblasts (HELF) compared with ASL from uninfected human skin as shown in Figure 4. ASL from patient extracts also caused significant apoptotic cell death comparable to 5 µg/ml of purified mycolactone after treatment of HELF for 48 h (Figure 5). No qualitative differences were observed in the physico-chemical properties, cytopathicity or pro-apoptotic activities of lipid extracts from the different forms of Mu infected lesions and there was no significant decline in percentage cytotoxicity between 5 untreated and 5 antibiotic treated lesions (Figure 4).

### Inhibition of induction of TNF-α release by J774 macrophages

**Figure 6:**
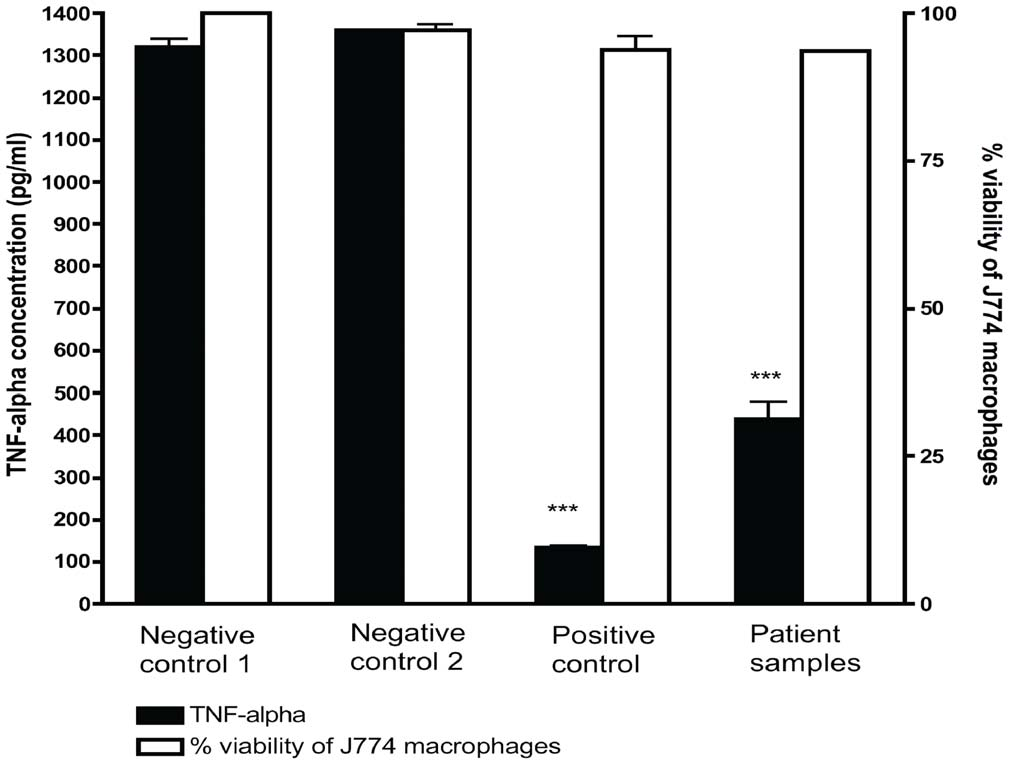
The effect of acetone soluble lipids from human Mu lesions on TNF-α release by J774 macrophages. J774 macrophages were stimulated with 0.5 µg/ml of LPS. Negative control 1 is untreated J774 macrophages, negative control 2 is J774 treated with ASL from uninfected skin. Positive control refers to purified mycolactone at a concentration of 500 ng/ml and patient samples refers to all 10 patient lesions. ASL from infected lesions significantly inhibited TNF-α release compared to both negative controls with ***p<0.001. Error bars are ±SEM of duplicate assays. Although TNF-α release by J774 macrophages was significantly inhibited by purified mycolactone and lipid extracts from patient lesions, this occurred without significant cytotoxicity.

**Figure 7:**
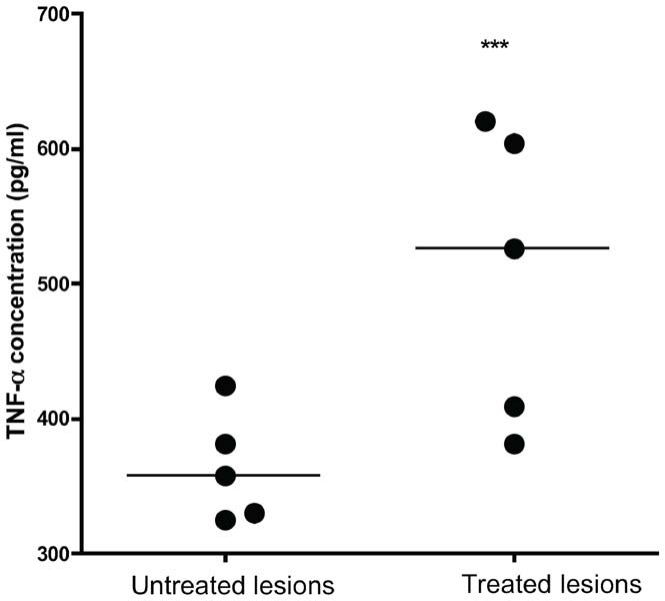
Comparison of TNF-α release by J774 macrophages treated with lipid extracts from 5 untreated and 5 antibiotic treated Mu infected human lesions. TNF-α was inhibited significantly more by lipid extracts from untreated lesions compared to antibiotic treated lesions. ***p<0.05 compared to untreated lesions. Lipid extracts from all lesions had detectable mycolactone signal on mass spectroscopic analysis.

Mycolactone is known to potently inhibit the release of cytokines at nanomolar, non-cytotoxic concentrations [16]. Figure 6 shows that ASL from infected human lesions significantly inhibited TNF-α release by murine macrophages compared with negative controls. There was no significant macrophage cytotoxicity despite the profound inhibition of cytokine release. Although there were no apparent differences in the degree of inhibition of TNF-α by lipid extracts from the different Buruli lesion types, significant recovery was observed in the release of TNF-α by macrophages treated with lipid extracts from antibiotic treated human lesions compared to untreated lesions (Figure 7) which may indicate a decline in tissue levels of mycolactone during antibiotic treatment.

## Discussion

Our primary objective in this study was to isolate mycolactone from infected human lesions. We have extracted lipids from infected human tissue using organic solvents and identified mycolactone A/B by its recognised physical properties on chromatography and mass spectrometry and by its biological activities such as cytotoxicity and immunosuppression. Our findings are the first definitive proof of the molecule's presence in infected human tissue and provide a foundation for further studies on the kinetics of mycolactone-mediated virulence.

Using ESI-MS, mycolactone A/B was detected in all 10 patient samples as an ion with an m/z of 765.5 [M+Na]^+^ and confirmed by its MSMS spectrum, the conserved core lactone ring and fatty acid side chain producing characteristic ions at m/z 429.3 and 359.2 respectively following fission of the ester bond with additional ions observed identical to those previously reported and identified in the MSMS spectrum of mycolactone A/B [19],[20].

Acetone soluble lipids from Mu infected lesions but not those from negative control human skin showed the typical phenotypic cytopathicity in cultured fibroblasts that is associated with mycolactone. Cytotoxicity was assessed by the ability of ASL from infected tissue to cause cytoskeletal re-arrangement, rounding up of cells and detachment from culture plates as well as inhibition of mitochondrial dehydrogenases. Furthermore TNF-α induction by LPS was significantly impaired by lipid extracts from infected human lesions but not by negative controls (Figure 6). These findings confirm that mycolactone in these clinical samples was biologically active both in its ability to cause cell death and to modulate immune responses. The data from these experiments provide the first direct evidence for the presence of mycolactone in Mu infected human skin tissue. Our observation that lipid extracts from human lesions induced cell death by both necrosis and apoptosis implicates mycolactone as the cause of the extensive tissue damage observed in humans with Mu disease.

The detection of mycolactone A/B in three common clinical forms of Mu lesions encountered in West Africa, namely nodular, oedematous and ulcerative, in this study re-emphasises its pivotal role in the pathogenesis of Mu disease. It is noteworthy that the molecule was detected throughout the course of disease from early nodular lesions to late ulcers. We also detected the molecule in lesions during the course of curative antibiotic therapy. Obiang et al in their landmark study reported that there was no correlation between mycolactone profiles of bacterial isolates from different patients and lesion type or severity [21]. These observations were not addressed in the present study but our findings open up the possibility that mycolactone concentration and type can be related to clinical presentation in future studies.

Mycolactone was not detected by TLC in two specimens that were AFB positive, one of which was also culture positive. This may be because the organisms were producing only small amounts of mycolactone, below the detection limit for TLC, or because the biopsies were taken from adjacent but different sites.

Figure 2B shows bands at rf 0.78 in most of the human samples but not in 9 and 10, and only weakly in 8. There was some variation in the weight and lipid content of specimens which might account for the different strength of non-mycolactone bands in human samples. Sample 8 showed a clear mycolactone band at rf 0.23 but only a very weak band at rf 0.78 suggesting that this sample contained a high concentration of mycolactone relative to the total lipid content. The purified mycolactone showed a band at rf 0.85 (Figure 2B) suggesting that it still contained some impurities but this does not affect the validity of the results.

The observation that LPS stimulated TNF-α release from murine macrophages was inhibited significantly more by lipid extracts from untreated lesions than by ASL from antibiotic treated lesions suggests that tissue mycolactone concentrations declined during antibiotic therapy. However the sample size was small and further prospective studies using quantitative mycolactone assays are needed to investigate the pharmacokinetics of tissue mycolactone and to relate them to clinical responses to antibiotic therapy and the recovery of systemic gamma interferon secretory responses we have observed [22]. The recent demonstration that mycolactone can be detected in circulating murine mononuclear cells [23] may prove to be pivotal in answering questions about the dynamics of the local and systemic immune response to Mu during antibiotic therapy.

In conclusion we have demonstrated for the first time the presence of mycolactone A/B in Mu infected human tissue. Given the central role played by mycolactone in the immunopathogenesis of Mu disease, it may be a useful marker for diagnosis and for assessment of the response to antibiotic therapy. Ultimately it may be possible to design molecules which can inhibit its synthesis or block its actions for the purpose of treatment.
